# Production and characterization of biocontrol fertilizer from brewer’s spent grain via solid-state fermentation

**DOI:** 10.1038/s41598-018-36949-1

**Published:** 2019-01-24

**Authors:** Lei Qiu, Jiao-Jiao Li, Zhen Li, Juan-Juan Wang

**Affiliations:** 1 State Key Laboratory of Biobased Material and Green Papermaking, Qilu University of Technology, Shandong Academy of Sciences, Jinan, Shandong 250353 PR China; 2 School of Biological Science and Technology, University of Jinan, Jinan, Shandong 250022 PR China

## Abstract

Brewer’s spent grain (BSG) is a promising substrate for the production of biocontrol fertilizer (BF). The effects of temperature, water content and fermentation time on the conidiation and germination rate of the entomopathogenic fungi *Beauveria bassiana* (BbQLU1) were modeled in a 3 × 3 × 3 factorially designed experiment. The optimum conditions for BF production (60% water content at 25 °C for 12 days) resulted in a conidiation of 0.85 × 10^8^ spores/g and a germination rate of 98.68%. BF at a concentration of 1 × 10^−2^ g/ml prompted plant growth and exhibited high toxicity against *Galleria mellonella* with an LT_50_ of 3.6 days. GC-MS analysis found 2-piperidone; benzoic acid, 3-methyl-, methyl ester; and other compounds to be potentially related to the toxicity and enhanced plant growth. These findings provide substantial evidence to support the production of BF.

## Introduction

Brewer’s spent grain (BSG) is a brewing byproduct available throughout the year at low cost and in large amounts. For each hectoliter of brew, 15–20 kg of wet BSG is produced^1^. The annual global production of BSG is estimated at 39 million tons^2^. BSG has traditionally been used as animal feed due to its high fiber and protein content as well as its low cost^2,3^. This use has proved beneficial; however, the Food and Drug Administration (FDA) proposed to restrict the utilization of BSG as animal feed^4^. Its supply can often exceed its demand^2^, and excess BSG is deposited in landfill^5^. BSG has therefore been utilized as a feedstock for the production of other value-added bioproducts such as lactic acid, ethanol and biogas^6–8^.

In recent years, demand for the production of biopesticides is an increasing global concern, because some synthetic pesticides damage the environment, food safety and health^9^. Additionally, pests cause global crop losses and develop high resistance to the chemical pesticides^10^. The wide expansion of biopesticides is consequently an alternative and permissible in integrated pest management as part of the National Organic Program^11,12^. The market in biopesticides is growing annually at a rate of 10–44%, depending on the country, e.g., the growth rate is 20% in Europe and Oceania^11^. More than 150 commercial products are available for use as biocontrol products of phytopathogens^13^.

Biopesticides are mainly becoming more accepted based on the advantages of entomopathogenic fungi, which are easy to cultivate, yield greater biomass and exhibit strong pathogenicity^14^. The substrate of rice can provide the essential nutrients for fungal growth via solid-state fermentation (SSF)^15,16^. Lowering the costs of substrates used for SSF has mainly focused on maximizing the yield of infective spores and enhancing their storage stability. The most used substrates are agroindustrial wastes such as coconut fiber, banana wastes, tea and coffee wastes, yucca wastes, and oil palm wastes^17–19^. The conidiation and germination of suitable fungal strains should also be considered in deciding SSF conditions, primarily the combination of moisture, temperature, and time^20^. The development of novel methods for optimizing both conidium production and germination are therefore essential for the supply of biopesticides based on *Beauveria bassiana* spores.

The purpose of the present work was to evaluate the feasibility of using BSG as a substrate for the production of biocontrol fertilizer (BF) via SSF employing the fungal strain BbQLU1. The specific process parameters for the production of BF were optimized by the surface response methodology. Additionally, the effective compounds and their effects on plant growth, which were assisted by BF, were explored.

## Results and Discussion

### Verification of the predicted optimal experimental conditions

When BSG was inoculated with BbQLU1 spores, the conidiation and germination rate fluctuated under the different treatments, indicating that temperature (A), water content (B), and time (C) have an interactive effect on the optimal conditions (Table s1). Three-dimensional graphs were generated for pair wise combinations of the three factors, with the third factor in each case fixed at its optimal level.

**Figure 1:**
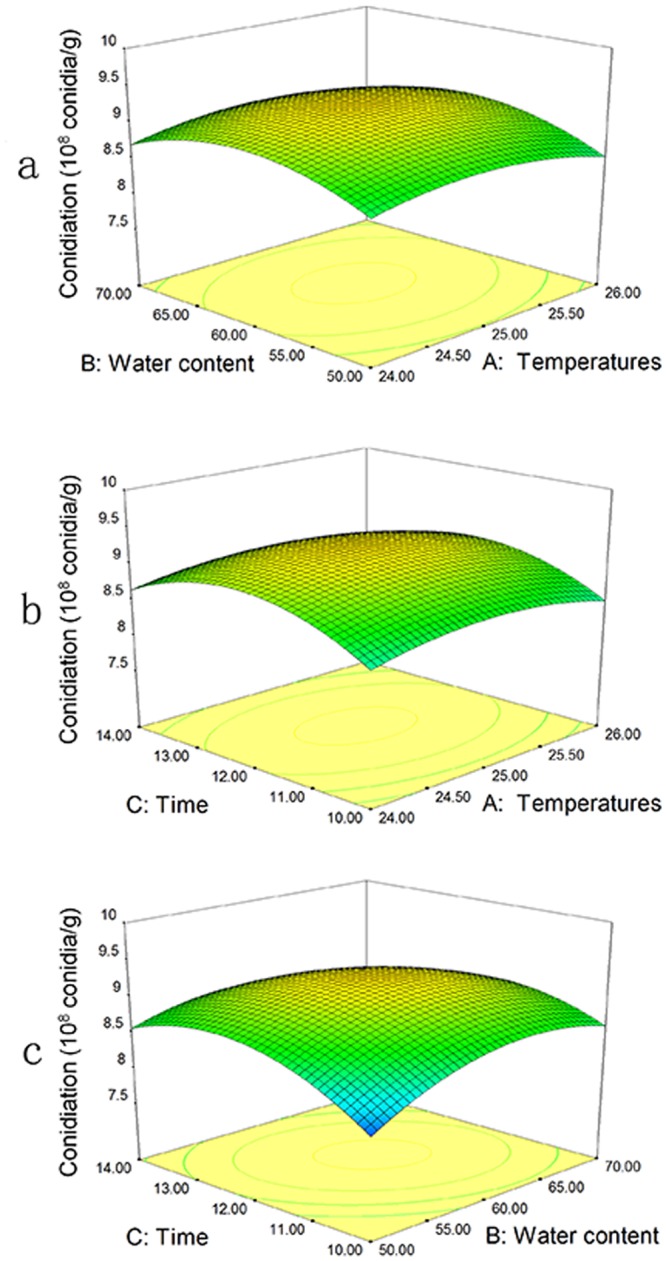
Response surface plots of conidiation of the strain BbQLU1. The interactions between (**a**) temperature and water content, (**b**) temperature and time, and (**c**) temperature and water content are shown.

With regards to conidiation, response surface methodology (Table 1, Eq. 1; Fig. 1) determined that the response and test variables were related by the following second-order polynomial equation:

1 $${\rm{Conidiation}}=9.24-0.014A+0.077{\rm{B}}+0.066{\rm{C}}+0.027{\rm{AB}}-0.020{\rm{AC}}-0.19{\rm{BC}}-0.22{{\rm{A}}}^{2}-0.38{{\rm{B}}}^{2}-0.48{{\rm{C}}}^{2}$$

**Table 1:** Compositions of the biocontrol fertilizer determined by GC-MS.

Peak	Retention time (min)	Molecular formula	Compound name	Molecular weight	Similarity index
1	7.900	C_8_H_24_O_4_Si_4_	Cyclotetrasiloxane, octamethyl-	296	96
2	10.733	C_10_H_30_O_5_Si_5_	Decamethylcyclopentasiloxane	370	95
3	11.700	C_5_H_9_NO	2-Piperidinone	99	97
4	11.833	C_10_H_8_	Azulene	128	94
5	11.950	C_11_H_16_	Diethylmethylbenzene	148	83
6	12.067	C_9_H_10_O_2_	Methyl 3-methylbenzoate	150	94
7	13.292	C_11_H_16_	Benzene, pentamethyl-	148	93
8	13.433	C_12_H_36_O_6_Si_6_	Cyclohexasiloxane, dodecamethyl-	444	93
9	13.625	C_11_H_10_	Naphthalene, 2-methyl-	142	96
10	13.875	C_11_H_10_	Naphthalene, 1-methyl-	142	96

From the ANOVA, the model F = 4.30, P(Prob > F) = 0.0338 (<0.05), indicating that the secondary model used in the experiment is statistically significant. An ANOVA revealed that the quadratic terms of water content (B^2^) and time (C^2^) significantly influenced conidiation (*P* < 0.05). The correlation measure for testing the goodness of fit of a regression equation is the adjusted determination coefficient, R^2^. Its value was only 0.8467, indicating that the correlation was not extremely high. In this case, the Lack of Fit F-value was 0.7220 (>0.05), which supports the model and the absence of Lack of Fit factors. The regression equation can therefore be used instead of the experimental points to analyze the experimental results, hinting that the effect of a specific experimental factor on response values is not a simple linear relationship. The fitted response for the above regression model, plotted in Fig. 1, demonstrates that the response surfaces for the two combinations were similar to each other. From the response surface in Fig. 1(c), fermentation time, compared to other variables, clearly had a significant effect on germination rate. The response surface diagram shows that within a certain range, the amount of sporulation is positively correlated with the temperature. When this range is exceeded, the amount of sporulation gradually decreases, indicating that the temperature increases to a certain value where sporulation peaks. The optimal temperature range, water content interval and fermentation time range were determined from Fig. 1 to be 24.47–25.47 °C, 53.38–63.38% and 11–13 days, respectively.

**Figure 2:**
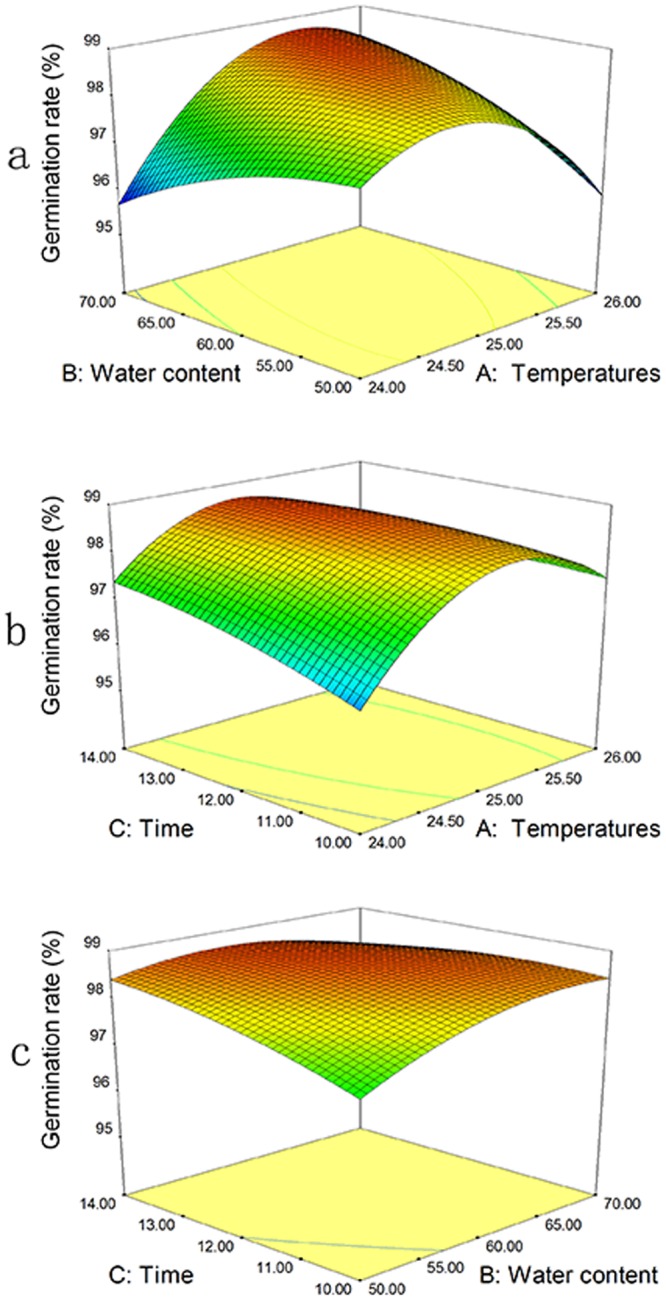
Response surface plots of the germination rate of the strain BbQLU1. The interactions between (**a**) temperature and water content, (**b**) temperature and time, and (**c**)temperature and water content are shown.

The second-order polynomial equation describing the germination rate (Table 1, Eq. 2; Fig. 2) was the following:

2 $${\rm{Germinationrate}}=98.62+0.27{\rm{A}}+0.16{\rm{B}}+0.23{\rm{C}}+1.05{\rm{AB}}-0.38{\rm{AC}}-0.35{\rm{BC}}-1.40{{\rm{A}}}^{2}-0.39{{\rm{B}}}^{2}-0.18{{\rm{C}}}^{2}$$

From the ANOVA, the model F = 11.64, P (Prob > F) = 0.0019 (<0.05), indicating that the secondary model used in the experiment is statistically significant. An ANOVA revealed that the interaction of temperature (A) and water content (B) significantly influenced the sporulation quantity (*P* < 0.01). The value of R^2^ was 0.9374, indicating that the correlation was extremely high. In this case, the Lack of Fit F-value is 0.0702 (>0.05), which supports the model and the absence of Lack of Fit factors. The regression equation can therefore be used instead of the experimental points to analyze the experimental results.

From the response surface in Fig. 2(a–c), temperature, compared to other variables, clearly had a significant effect on the germination rate. The response surface curve shows that the minimum temperature at which conidial germination occurred was 10 °C. Fig. 2(a,b) shows that increases in temperature from 24 to 25 °C were accompanied by increases in conidial germination, followed by a gradual decrease in the percentage of germinated conidia as the temperature further increased from 25 to 26 °C. Higher percentages of germinated conidia were obtained by varying the concentration of water and the fermentation time (Fig. 2(c)), which demonstrates that the response surfaces for the two combinations were similar to each other. The effect of temperature on spore germination rate is more distinct than the effects of water content and fermentation time. The optimal temperature, water content, and fermentation time ranges were determined from Fig. 2 to be 24.5–25.5 °C, 58.29–63.29%, and 11.5–13.2 days, respectively.

The development of processes to produce *B*. *bassiana* has primarily focused on lowering cost by maximizing the yield of effective conidial preparations. Some agro-industrial wastes such as rice straw, wheat bran, and grains of millet have been used as solid substrates for *B*. *bassiana* conidium production^16,21^. However, the use of BSG for BF production through the fermentation of *B*. *bassiana*, an entomopathogenic fungus, has not been reported. BSG is usually full of rich starch and fibre that can facilitate B. bassianaconidium germination and mycelial growth^7,22^. BSG has rich inert lignocellulose, which can retain water and increase the porosity in the substrate while maintaining a supply of moisture to promote fungal growth^23,24^. We used discarded BSG as the main raw material in this study to improve the hydrophobicity in SSF. Hydrophobicity is strikingly important in solid state culture^25^. The radial extension rate was somewhat considered an estimation of fungal ability to colonize the substrate on which it grows^26^. The radial extension rates were markedly enhanced after the addition of liquid mediaor standard Sabouraud Dextrose broth^27^.

The conditions that produced *B*. *bassiana* conidia should be considered for maintaining high levels of germination and constant quality while maximizing production^15^. The optimal fermentation conditions are temperature of 25.10 °C, water content of 61.55%, and time of 12.17 days, which resulted in a spore germination rate of 98.68% and conidiation of 0.85 × 10^8^ spores/g. Furthermore, several investigators have reported that the optimum temperatures for *B*. *bassiana* mycelial growth, conidial germination, sporulation, and virulence are in the range from 20–30 °C^21–28^. Two optimized parameters were a 40% moisture content and 25 °C as the culture temperature when grown in a solid-state culture using rice as the *B*. *bassiana* substrate^29^. As another example, the optimum temperature for conidial germination and vegetative growth in several strains of *B*. *bassiana* is approximately 25 °C^30^.

### Biocontrol efficacy of BF

**Figure 3:**
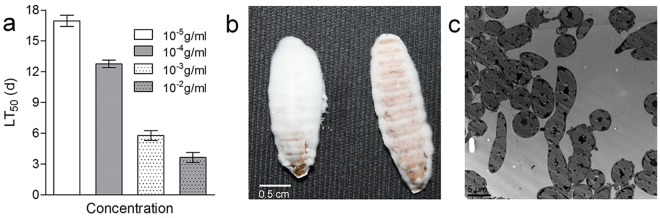
Influence of biocontrol fertilizer on *Galleria mellonella* larvae following treatment with different concentrations (**a**) Median lethal time (LT_50_) for biocontrol fertilizer against *G*. *mellonella* larva (F_3,8_ = 509.5300, p < 0.001), (**b**) *G*. *mellonella* larvae treated with biocontrol fertilizer for 8 days, and (**c**) the *B*. *bassiana* in the *G*. *mellonella* larval tissues under an electron microscope are shown.

The system of *G*. *mellonella* infection was used to bioassay the virulence of the BF. The results indicate that the concentration of BF remarkably affected the *G*. *mellonella* larvae mortality (Fig. 3, F_3,8_ = 509.5300, p < 0.001). Analyses of virulence by BF against *G*. *mellonella* larvae by cuticle penetrationat 1 × 10^−2^ g/ml (containing the 0.8 × 10^6^spores) resulted in an LT_50_ of 3.2–4.1days (Fig. 3a). There was no significant difference in insecticidal effect, compared with the same concentration of spores cultured on SDAY. Based on the LT_50_ means, the mortality was 1.6-, 3.5, and 4.7-fold slower than this ratewhen treatments of 1 × 10^−3^, 1 × 10^−4^, and 1 × 10^−5^g/ml, respectively, were applied. All these estimates implicated significant virulence against *G*. *mellonella* larvae at all tested concentrations of BF. When the dead *G*. *mellonella* larvae were cultivated in moisturized filter paper for 2 days, their bodies gradually shrank and became black-gray. In addition, they were soon covered with white and fluffy mycelia, hinting that they had become infected and killed by *B*. *bassiana* spores (Fig. 3b). Microscopic observation of the dead larva clearly distinguished blastospores on the basis of size and morphology (Fig. 3c).

Previous studies have reported that the virulence of entomopathogenic fungi is affected by biotic and abiotic factors^31^.These biotic factors include the isolated fungus, the target species and another host. The pathogenicity of identified *B*. *bassiana* isolates was evaluated at a concentration of 10^9^ conidia/ml against the *G*. *mellonella*, yielding a lethal time (LT_50_) of 1.7days^32^. On the other hand, the biotic factors primarily depend on environment conditions such as humidity and temperature in controlling post-harvest insects^33,34^.

### The effect of the BF on plant growth

**Figure 4:**
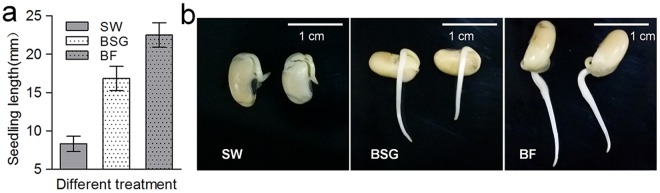
Effects of 24 h of treatment of different seed inoculants (SW: sterile water, BSG: brewer’s spent grain, and BF: biocontrol fertilizer) on mean (±SE) seedling height. (**a**) A column diagram (F_2,6_ = 75.592; P < 0.001) and (**b**) a picture of grown plants are shown. Bars with different letters differ significantly from all seed treatment durations at the *P* = *0*.*05* level (Duncan’s Multiple Range Test after two-way ANOVA).

The effect of the BF on plant growth was determined by measuring root length, which was measured from the soybean surface to the tip of the sprout. Both BSG and BF had significant effects on almost all of the seedling growth parameters sampled 24 hours post-inoculation in both experimental replicates (Fig. 4a). The average seedling growth with sterilized water was 8.3 ± 0.6 mm; this value was 16.8 ± 0.9 mm when the sample was treated with BSG and, more significantly, 25.2 ± 0.9 mm when the sample was treated with BF. Treatment with BF significantly enhanced the percent seedling length (F_2,6_ = 75.592; P < 0.001), indicating that this experiment generated an extremely significant difference. Figure 4(b) shows that the root length of soybean was twice as long in the sample treated with BSG than that treated with sterile water and that the root length was 50% greater in the sample treated with BF than that treated with BSG. BSG is a novel rich and low-cost source of fiber and protein^22^, so the seedling can substantially absorb nutrients to increase the length of its root.

The root growth of seedlings is closely related to microgradients in the BF. These microgradients are involved in not only the rich BSG but also plant growth promoters^35,36^. In addition, they can directly affect colonization by mycelia and conidiation^37^. On the other hand, BF affected seedling growth more positively than BSG in our study. The tested endophyte *B*. *bassiana* plays a key role in growth promotion and productivity in BF^38^. Similar reports of plant growth promotion by endophytic fungal entomopathogens have been documented. Inoculation of wheat plants with *B*. *bassiana* has a significant impact, causing their root weight to become much greater than the root weight of control plants^39^.Entomopathogenic fungi can produce “siderophores” in soil to promote plant growth by increasing the pH value of the culture medium^40,41^. Furthermore, *B*. *bassiana* can colonize not only roots but also stems and leaves^42,43^. According to related literature, BF contains not only active *B*. *bassiana* microorganisms but also the metabolic pathways related to various organic substances. *B*. *bassiana* is present in roots to promote root growth, showing that greater speed and colonization are closely related. Several different endophytic fungi that exhibit tissue specificity possess have somewhat adapted to particular conditions present inside specific plant tissues. Many studies have reported that different fungi have varying ability to colonize different tissues of plants. For instance, *Metarhizium anisopliae*ICIPE 20 is seriously affected by the bean stem maggot, demonstrating that fungal endophytes can be promising tools for management^44^. Moreover, *B*. *bassiana* has been detected in both old and young stem/leaf tissuesin the fungus-treated maizeplants^45^. The tested benefits of BF on plant growth and nutrition to provide support for more sustainable biocontrol agents is thus currently underway.

### Identification of the effective compounds in the BF

**Figure 5:**
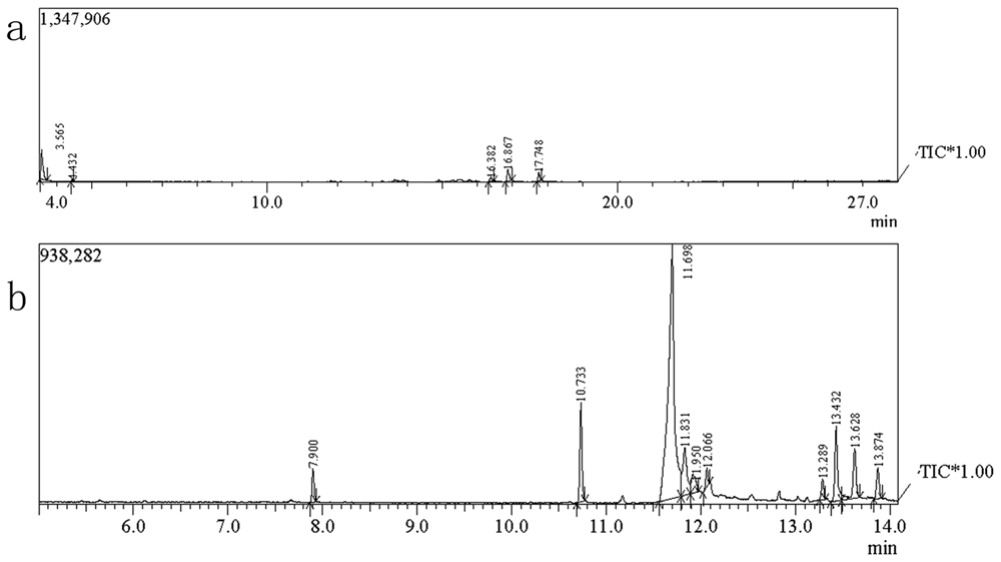
Total ion chromatograms for the gas chromatography-mass spectrometry analysis of (**a**) BSG and (**b**) BF.

Identification of an unknown component in BF usingGC-MS depends on its retention time and mass spectra. Through GC-MS analysis, we were able to identify 10 low-molecular-weight polar metabolites, which are not easily determined by an LC-MS approach, using the unknown samples (Fig. 5a) BSG and (Fig. 5b) BF, respectively (Table 1).

The chemical constituents were identified by GC-MS, and the chemical components detected in peaksIII and VI could be insecticidally effective substances. The position of peak III, whose relative content was higher than that of peak VI, was 11.698 min, and this compound was identified as 2-piperidone by the GC-MS mass library. The position of peak VI, whose relative content was low, was 12.066 min, and this compound was identified as methyl 3-methylbenzoate by the GC-MS mass spectrometry library.

Several entomopathogenic fungus toxic bioactive compounds such as small secondary metabolites, cyclic peptides and macromolecular proteins can be purified^46^. For example, cyclicpeptides with immunosuppressive properties, cyclosporins A and C, were detected in *B*. *bassiana* mycelia, have an insecticidal effect on mosquito larvae and induce cuticular blackening^47,48^.An insect toxin protein, Bb70p, which was recently purified from *B*. *bassiana* 70, caused high mortality in *G*.*mellonella* and demonstrated a potential to enhance virulence^49^. Although 2-piperidone and methyl 3-methylbenzoate are shown above to be toxic components, their roles remain poorly understood. In the present study, the GC-MS mass spectra of BF have many small spectrogram peaks that are absent from the spectra of BSG, hinting that *B*. *bassiana* synthesized other unknown metabolites. Although very little is known about the secretion of bioactive metabolites by *B*. *bassiana* during growth metabolism or infection, we acknowledge that BF potentially increases plant growth and controls pests. The combination of pest management strategies currently requires the use of safe and environmentally sound biological control agents, so it is more efficient and cost effective than the fungicides in controlling pests.

## Conclusions

This study is first to use BSG to produce BF through the fermentation of *B*. *bassiana*. BSG is usually full of rich starch and fibre that can facilitate B. bassiana conidium germination and mycelial growth. This experiment optimizes the culture conditions by the response surface analysis method, which not only optimizes the main factors controlling SSF and shortens the testing period but also effectively evaluates the interactions between factors. The optimum conditions for BF production are following the 60% water content at 25 °C for 12 days. In addition to the main factors of temperature, water content and fermentation time, the oxygen concentration, osmotic pressure and other factors will also alter the effects of BF^50–53^, and their ranges should thus be considered.

BSG enhances the nutritional value of BF, which facilitates conidial germination and speeds. Applications of BF on the *G*. *mellonella* larvae can be an effective and efficient biological control strategy for the management of soil-born insects. BF affected seedling growth more positively than BSG in our study. The tested endophyte *B*. *bassiana* may plays a key role in growth promotion and productivity in BF. Through GC-MS analysis, we were able to identify 10 low-molecular-weight polar metabolites, in which 2-piperidone and methyl 3-methylbenzoate could be insecticidally effective substances.

These results elucidate the production and function of BF, and add the new knowledge to what are known about the virulence and potential promotion of bioagent. Such theory and technique would be helpful for enriching the type of fertilizer and the biocontrol of soil-borne plant pathogens, which can propell the future industrialization of microbial fertilizers forward.

## Methods

### Test strains

The fungal strain BbQLU1 (China General Microbiology Culture CollectionCenter–CGMCC; Accession No. 11618) was cultured at 25 °C on Sabouraud dextrose agarplus yeast extract (SDAY, 4% glucose, 1% peptone, 1.5% agar and 1% yeast extract) medium for normal growth.

### BSG

The BSG samples were collected from the Tsingtao Brewery located in Jinan, China, and stored at −20 °C until they were used. The fresh BSG was presented the following composition (w/w): 70.16% water, 16.92% nitrogen-free extract, 8.48% crude protein, 1.53% crude fat and 1.34% crude fiber. As soon as obtained, the material was dried at 50 ± 5 °C until approximately 15% moisture content. The BSG was not milled before pretreatment.

### Inoculation of BSG and experimental design

Cultures of BbQLU1 were prepared by shaking 50 ml of SDAY at a concentration of 10^4^ conidia per milliliter in 250 ml flasks at 25 °C for 3days, followed by inoculation. The combination of the cultures and the BSG was incubated under the different conditions listed in Table s1. The experimental design was a 3 × 3 × 3 factorial that includedthree fermentation temperatures, three water contents and three fermentation times. In total, 17 runs were performed as apart of the experimental design. The parameters of fermentation temperature, water content and fermentation time were chosen as the main variables and designated A, B, and C, respectively. Low, middle and high values of fermentation temperature, water content and fermentation time were designated −1, 0, and 1, respectively. The water content of BSG was adjusted by adding sterilized distilled water. The fermentations of BSG were incubated on plates (9 cm diameter) and incubated for 10 days. All experiments were performed in triplicate, and the average values of conidiation and germination rates were used to select the optimal conditions.

### Assessment of conidiation capacity and germination rate

Conidia on each plate were washed off in 100 ml of 0.02% Tween 80 by 10 min of vibrations. Conidial concentrations were determined in the suspension by using microscopic counts with a hemocytometer and converted to the number of conidia pergramas an index of conidial yield. The conidia cultured on BSG were suspended at a concentration of 10^6^ conidia/ml using 0.5% Tween-80 sterile solution. Next, 50 μL aliquots of conidial suspension was spread onto plates (9 cm) of GM (2% sucrose, 0.5% peptone and 1.5% agar), and the plates were incubated for 24 h at 25 °C and in a 12:12 h cycle. From 4 h onwards, the percent germination on each plate was assessed every 2 h using three microscopic counts.

### Assessment of the virulence of conidia and the BF

*G*. *mellonella* infected by topical application was used to bioassay the virulence of BF via cuticle penetration. Briefly, batches of 30–40 *G*. *mellonella* larvae (~300 mg per larva) were immersed for ~10 s in 30 ml of 1 × 10^−5^, 1 × 10^−4^, 1 × 10^−3^,and 1 × 10^−2^ g/ml suspension with BF (treatment) or BSG with sterile distilled water (control). Each batch of the immersed larvae were incubated in Petri dishes (15 cm diameter) for 35 days at 25 °C and examined at 12 h intervals for mortality records. The median lethal time (LT_50_, days) of each strain against *G*. *mellonella* larvae was estimated by probit analysis of time-mortality trends from the bioassays. All the values and estimates from three repeated assays of the tested strains were analyzed via an analysis of variance (ANOVA).

### The effect of BF on soybean growth

Surface-sterilized soybean seeds were immersed in 60% alcohol solution for 10 min and treated with sterile distilled water, BSG (0.01 g/ml), or BF (0.01 g/ml) for 8 h (n = 30 for each treatment). The seeds were maintained in the incubators at 25 °C for the designated duration. All experiments were performed in triplicate. Each batch of the immersed seeds was incubated in Petri dishes (15 cm diameter) at 25 °C, and their root lengths were examined.

### GC-MS analysis

BF (1 g) was immersed in 100 ml of sterile water for 12 h, and the mixture was then centrifuged at 5000 r/min for 15 min. After that,100 ml of 75% ethanol was added, and the sample was allowed to precipitate for 24 h at room temperature. The sample was vortexed and then centrifuged at 5000 r/min for 15 min. The upper layer of the sample was added to twice its volume of ethyl acetate to soak overnight, and the upper, organic phase was taken in layers. The lower, aqueous phase was washed with twice its volume of ethyl acetate. This extraction procedure was repeated three times.The combined fractions of the upper, organic-phase ethyl acetate were concentrated under reduced pressure. The crude extract of the toxin was eluted by thin-layer chromatography to obtain pure toxin. After the toxin had been diluted with twice its volume of ethyl acetate, the concentrated solution was filtered through a 0.45-μm filter to obtain sample for spotting. The injection volume was 1.0 μL, and injections were made in splitless mode. The following oven temperature program was used with helium as the carrier gas: the temperature was maintained at 50 °C for 3 min, increased to 250 °C at a ramp rate of 10 °C/min and then held for 5 min, giving a total run time of 28 min. Electron impact ionization mode was used for ionization. The ionizing energy was 70 eV. Qualitative analysis was conducted in full scan mode (m/z range: 20–650).GC-MS data analysis was conducted by integrating eachresolved chromatogram peak. A 2-piperidone standard (purity 98%) was verified on a gas chromatograph to elute at 11.609 min. The results showed that the peak time for the standard and peak III was approximately the same. A methyl 3-methylbenzoate standard (purity 99%) was verified on a gas chromatograph to elute at 12.042 min. Identification of toxic components was performed by searching for mass spectra using the NIST08 and the Golm (http://gmd.mpimp-golm.mpg.de/) libraries and when available, by comparison with analytical standards.

### Statistics

Differences between the results from treatments were calculated and statistically analyzed using ANOVA and Box-Behnken tests (*P* < 0.05). Surface response methodologywas applied to evaluate the effects of the three factors on BbQLU1conidiation and germination rate. The parameters oftemperature, water content and time were chosen as the mainvariables and designated A, B and C, respectively. Low, middleand high values oftemperature, water content and time were designated −1, 0 and 1, respectively. Response surface models were constructedusing Design-Expertsoftware(trial version 8.0.5.0; Stat-Ease, Inc.,Minneapolis, MN, USA).

## Supplementary information

Table s1
