# Supplementary material for: Production and characterization of biocontrol fertilizer from brewer’s spent grain via solid-state fermentation
Source: Sci Rep. 2019 Jan 24;9:480. doi: 10.1038/s41598-018-36949-1 (PMC6345932; doi:10.1038/s41598-018-36949-1)
Supplement: Supplementary file 1 — Table s1 [file 41598_2018_36949_MOESM1_ESM.docx]

**Supplementary Material**

**Production and characterization of biocontrol fertilizer from brewer’s spent grain via solid-state fermentation**

Lei Qiu ^1^ *, Jiao-Jiao Li ^1^ *, Zhen Li ^1^ *, Juan-Juan Wang ^2^

* These authors equally contributed to this work.

Correspondence and requests for materials should be addressed to J.J.W. (e-mail: [wjj880414@163.com](mailto:wjj880414@163.com)) or L.Q.(e-mail: [qiulei.2005@163.com](mailto:qiulei.2005@163.com))

1. State Key Laboratory of Biobased Material and Green Papermaking, Qilu University of Technology, Shandong Academy of Sciences, Jinan, Shandong 250353, PR China

2. School of Biological Science and Technology, University of Jinan, Jinan, Shandong 250022, PR China

**Table s1** Factorial design of variables with *B. bassiana* conidiation and germination rate as responses.

| **Run** | **Temperature**  **(°C)** | **Water**  **content (%)** | **Time**  **(D)** | **Conidiation**  **(10^8^ conidia/g)** | **Germination rate (%)** |
| --- | --- | --- | --- | --- | --- |
| 1 | 24 | 70 | 12 | 8.63 | 95.85 |
| 2 | 26 | 60 | 10 | 8.57 | 97.86 |
| 3 | 25 | 70 | 10 | 8.61 | 97.96 |
| 4 | 25 | 60 | 12 | 9.32 | 98.65 |
| 5 | 26 | 60 | 14 | 8.53 | 96.94 |
| 6 | 26 | 70 | 12 | 8.62 | 98.31 |
| 7 | 24 | 60 | 14 | 8.56 | 96.98 |
| 8 | 25 | 60 | 12 | 9.62 | 98.91 |
| 9 | 25 | 60 | 12 | 9.23 | 98.67 |
| 10 | 25 | 60 | 12 | 9.16 | 98.63 |
| 11 | 24 | 50 | 12 | 8.71 | 97.47 |
| 12 | 25 | 50 | 10 | 7.89 | 97.08 |
| 13 | 26 | 50 | 12 | 8.59 | 95.72 |
| 14 | 25 | 50 | 14 | 8.54 | 98.85 |
| 15 | 25 | 60 | 12 | 8.87 | 98.26 |
| 16 | 24 | 60 | 10 | 8.52 | 96.38 |
| 17 | 25 | 70 | 14 | 8.49 | 98.32 |
